# Digital Spatial Profiling identifies distinct patterns of immuno-oncology-related gene expression within oropharyngeal tumours in relation to HPV and p16 status

**DOI:** 10.3389/fonc.2024.1428741

**Published:** 2024-09-12

**Authors:** Jill M. Brooks, Yuanning Zheng, Kelly Hunter, Benjamin E. Willcox, Janet Dunn, Paul Nankivell, Olivier Gevaert, Hisham Mehanna

**Affiliations:** ^1^ Institute of Head and Neck Studies and Education, Institute of Cancer and Genomic Sciences, University of Birmingham, Birmingham, United Kingdom; ^2^ Stanford Center for Biomedical Informatics Research (BMIR), Department of Medicine, and Department of Biomedical Data Science, Stanford University, Stanford, CA, United States; ^3^ Propath, Hereford, United Kingdom; ^4^ Institute of Immunology and Immunotherapy, College of Medical and Dental Sciences, University of Birmingham, Birmingham, United Kingdom; ^5^ Warwick Clinical Trials Unit, University of Warwick, Coventry, United Kingdom; ^6^ National Institute for Health and Care Research (NIHR) Birmingham Biomedical Research Centre, University of Birmingham, Birmingham, United Kingdom

**Keywords:** oropharyngeal cancer, Human Papilloma Virus (HPV), p16, dual p16/HPV testing, treatment de-intensification, Digital Spatial Profiling, tumour microenvironment

## Abstract

**Background:**

The incidence of oropharyngeal cancer (OPC) is increasing, due mainly to a rise in Human Papilloma Virus (HPV)-mediated disease. HPV-mediated OPC has significantly better prognosis compared with HPV-negative OPC, stimulating interest in treatment de-intensification approaches to reduce long-term sequelae. Routine clinical testing frequently utilises immunohistochemistry to detect upregulation of p16 as a surrogate marker of HPV-mediation. However, this does not detect discordant p16-/HPV+ cases and incorrectly assigns p16+/HPV- cases, which, given their inferior prognosis compared to p16+/HPV+, may have important clinical implications. The biology underlying poorer prognosis of p16/HPV discordant OPC requires exploration.

**Methods:**

GeoMx digital spatial profiling was used to compare the expression patterns of selected immuno-oncology-related genes/gene families (n=73) within the tumour and stromal compartments of formalin-fixed, paraffin-embedded OPC tumour tissues (n=12) representing the three subgroups, p16+/HPV+, p16+/HPV- and p16-/HPV-.

**Results:**

Keratin (multi KRT) and *HIF1A*, a key regulator of hypoxia adaptation, were upregulated in both p16+/HPV- and p16-/HPV- tumours relative to p16+/HPV+. Several genes associated with tumour cell proliferation and survival (*CCND1*, *AKT1* and *CD44*) were more highly expressed in p16-/HPV- tumours relative to p16+/HPV+. Conversely, multiple genes with potential roles in anti-tumour immune responses (immune cell recruitment/trafficking, antigen processing and presentation), such as *CXCL9*, *CXCL10*, *ITGB2*, *PSMB10*, *CD74*, HLA-DRB and *B2M*, were more highly expressed in the tumour and stromal compartments of p16+/HPV+ OPC versus p16-/HPV- and p16+/HPV-. CXCL9 was the only gene showing significant differential expression between p16+/HPV- and p16-/HPV- tumours being upregulated within the stromal compartment of the former.

**Conclusions:**

In terms of immune-oncology-related gene expression, discordant p16+/HPV- OPCs are much more closely aligned with p16-/HPV-OPCs and quite distinct from p16+/HPV+ tumours. This is consistent with previously described prognostic patterns (p16+/HPV+ >> p16+/HPV- > p16-/HPV-) and underlines the need for dual p16 and HPV testing to guide clinical decision making.

## Background

Head and neck cancer (HNC) is the seventh most common cancer worldwide (1). Incidence rates are rising; mostly driven by a rapid increase in oropharyngeal cancer (OPC) incidence within certain global regions including the United States (US), Europe, New Zealand, and parts of Asia (2–4). The main risk factors for OPC include smoking, excessive alcohol intake and infection with high-risk Human Papilloma Virus (HPV). It is the increase in the latter which underpins rising incidence rates. In the US and United Kingdom, HPV-mediated OPC is now more prevalent than HPV-mediated cervical cancer (3, 5). It is estimated that incidence will continue to rise for the next ~20 years before the impact of gender-neutral prophylactic vaccination is felt (6, 7).

HPV-mediated OPC has distinct epidemiological, molecular, and immunological features compared with HPV-negative disease and is associated with better treatment response and outcomes (8, 9). This has led to separate classifications in the latest UICC/AJCC TNM staging system (TNM8) (10). Given the improved prognosis (8) and younger age of HPV-positive OPC patients, there is considerable interest in approaches to de-intensify treatment and reduce long-term morbidity and quality-of-life impact. Unfortunately, clinical trials to date have reported limited or no success (11, 12). One potential explanation for this relates to the determination and definition of HPV status.

The presence of HPV in OPC can be assessed directly using PCR-based methods or *in situ* hybridisation to detect viral DNA/RNA or indirectly using immunohistochemistry (IHC) to assess overexpression of the protein p16^INK4a^ (p16) (13, 14). p16 overexpression is the indirect result of HPV early protein 7 (E7)-mediated inactivation of retinoblastoma protein. In contrast, frequent loss, mutation, or epigenetic silencing of the *CDKN2A* gene encoding p16 results in low/absent expression in HPV-negative OPC. Detection of p16 overexpression is therefore a good surrogate marker for HPV status and – being simple and cost-effective to implement – is routinely used in clinical practice. However, dual p16 and HPV-DNA/RNA testing has recently shown that while the majority of p16-positive tumours are HPV-positive (p16+/HPV+), a subset (~10%) are HPV-negative (p16+/HPV-). Likewise, a small subset of HPV-positive tumours do not overexpress p16 (p16-/HPV+). An important issue is whether these two discordant subsets – particularly those that are p16+/HPV- and therefore assigned as ‘HPV-positive’ by p16 routine testing – share the improved treatment response and survival outcomes of p16+/HPV+ cases, or whether outcomes are more closely aligned with p16-/HPV- tumours. Multiple studies suggested differential prognosis but were limited by sample size or restricted geographical sampling (15–19). A recent large multicentre study (n = 7,654) provided strong evidence that patients with discordant OPC (p16+/HPV- or p16-/HPV+) have significantly worse prognosis compared with p16+/HPV+ OPC patients, although significantly better than p16-/HPV-patients (20).

The biology underpinning this intermediate outcome requires exploration to develop understanding, guide development of novel therapies and inform clinical decision making. Here, we utilised NanoString’s GeoMx Digital Spatial Profiling (DSP) platform to explore *in situ* differences in gene expression between p16+/HPV+, p16+/HPV- and p16-/HPV- oropharyngeal tumours. This approach enables spatially-resolved analysis of gene expression within defined regions of interest, selected based on expression of morphology markers; for example, pan-cytokeratin expression to identify tumour versus stroma in epithelial tumours and/or CD3 to identify T cell rich areas.

## Methods

### Cohort

The study utilised formalin-fixed, paraffin-embedded (FPPE) diagnostic biopsy samples (primary tumour) from 12 OPC patients (four p16+/HPV+, three p16+/HPV- and five p16-/HPV-) recruited to the PET-NECK (21) or Predictr (22) clinical studies between 2000 and 2012. Patients were treated with curative intent, with either platinum-based chemoradiotherapy or surgery followed by adjuvant radiotherapy/chemoradiotherapy. Ethical approval for use of tissue samples in translational research was granted by the North West - Preston Research Ethics Committee (Reference: 16/NW/0265).

p16 status was assessed by immunohistochemical staining using the CINtec Histology kit (Roche). Samples with strong nuclear and cytoplasmic staining in >70% of the tumour were considered positive. HPV status was determined by DNA *in situ* hybridisation using the Ventana INFORM HPV III Family 16 probe or HPV DNA PCR.

### Digital spatial profiling

5μm tissue sections were cut and mounted on SuperFrost plus microscope slides. Digital Spatial profiling (DSP) was carried out at NanoString Technologies Inc. Seattle, USA as part of their Technology Access Programme and according to their standard protocol (23). Briefly, slides were hybridised/stained with oligo-conjugated RNA detection probes [immuno-oncology panel comprising 78 genes, 73 target genes and five controls (**Table 1**)], plus three fluorescent conjugated antibodies and nuclear stain for characterisation of tissue compartments to facilitate Region of Interest (ROI) selection. ROIs were segmented into multiple regions representing tumour and stromal (non-tumour) tissue, using a threshold classifier on a fluorescently labelled pan-cytokeratin (PanCK) stain (clone AE1/AE3 594 Novus, 1:200 dilution). Fluorescent antibodies to CD3 (UMAB54 647 Origene, 1:100), to identify T cells, and CA-IX (EPR1451(2) 488, AbCam, 1:100), as a marker of hypoxia, were included to aid ROI selection, with nuclear stain (Syto 83 532, ThermoFisher, 1:25). Slides were imaged and a total of 141 ROIs across the 12 samples selected for analysis. ROIs were then sequentially illuminated with UV light to cleave oligo-probes which were aspirated and dispensed into 96-well plates. Probes were then hybridised to optical barcodes and counted using the nCounter platform (NanoString Technologies). Digital raw counts were exported for analysis.

**Table 1:** Target genes included in the GeoMx Immuno-oncology human RNA panel.

Immune cell typing/ profiling	Immune activation status	Antigen presentation	Immune checkpoints & drug targets	Cytokine & chemokine signalling	Cell adhesion & migration	Apoptosis	Tumour markers & signalling	Proliferation	Reference genes
*BATF3* *CD3E* *CD4* *CD8A* *CD47* *CD68* *FOXP3* *LY6E* *MS4A1* *PTPRC* *TBX21*	*CD27* *CD40LG* *CD44* *CD86* *GZMB* *ICOSLG* *NKG7* *TNFRSF9*	*B2M* *CD74* HLA-DRB HLA-DQ *HLA-E* *PSMB10*	*ARG1* *CD274* *CD276* *CTLA4* *HAVCR2* *IDO1* *LAG3* *PDCD1* *PDCD1LG2* *TIGIT* *VSIR*	*CCL5* *CMKLR1* *CSF1R* *CXCL10* *CXCL9* *CXCR6* FAS* HIF1A* *IFNAR1* *IFNG* *IFNGR1* *IL12B* *IL15* *IL6* *STAT1* *STAT2* *STAT3* TNF* *VEGFA*	*ICAM1* *ITGAM* *ITGAV* *ITGAX* *ITGB2* *ITGB8* *PECAM1*	*BCL2* FAS* TNF*	*AKT1* *CTNNB1* *DKK2* *EPCAM* HIF1A* multi KRT^#^ pan-melanocyte *PTEN*	*CCND1* *MKI67*	*OAZ1* *POLR2A* *RAB7A* *SDHA* *UBB*

*genes included in dual categories.

^#^detects multiple keratin types, hereafter referred to as KRT.

### Data analysis

Quality control and normalisation were performed in accordance with NanoString’s Gene Expression Data Analysis Guidelines MAN-C0011–04, 2017 (https://nanostring.com/wp-content/uploads/Gene_Expression_Data_Analysis_Guidelines.pdf). nCounter readout performance was assessed by evaluating the imaging and binding density QC metrics. All imaging segments demonstrated a high percentage of successfully scanned subsections, with a registered Fields of View (FOV) above 88%. The binding density was below 0.58, indicating low competition for binding the flow cell. Gene expression counts for the target genes were normalised to a set of house-keeping genes (*RAB7A*, *UBB*, *SDHA*, *POLR2A*, *OAZ1*), and a normalisation factor was calculated for each segment by comparing the geometric means of house-keeping genes across all segments. The normalised gene expression data were imported into Anndata objects (v0.8.0), and the gene expression values log2 transformed. Differential gene expression analysis was performed using the Kruskal-Wallis test, and pairwise comparisons were made using the Wilcoxon rank-sum test using the Scanpy library (v1.9.6). Raw p-values were adjusted using the Benjamini-Hochberg procedure. To generate the heatmaps, we selected genes that were significantly upregulated in one group compared to the other groups. For this, we required the raw p-values to be smaller than 0.05 and the adjusted p-values to be smaller than 0.2. To generate the volcano plots, we performed pairwise comparisons between each pair of groups, and the significant genes were determined using adjusted p-values below 0.05. Volcano plots were generated using the ggplot2 R library (v3.5.1).

## Results

**Figure 1:**
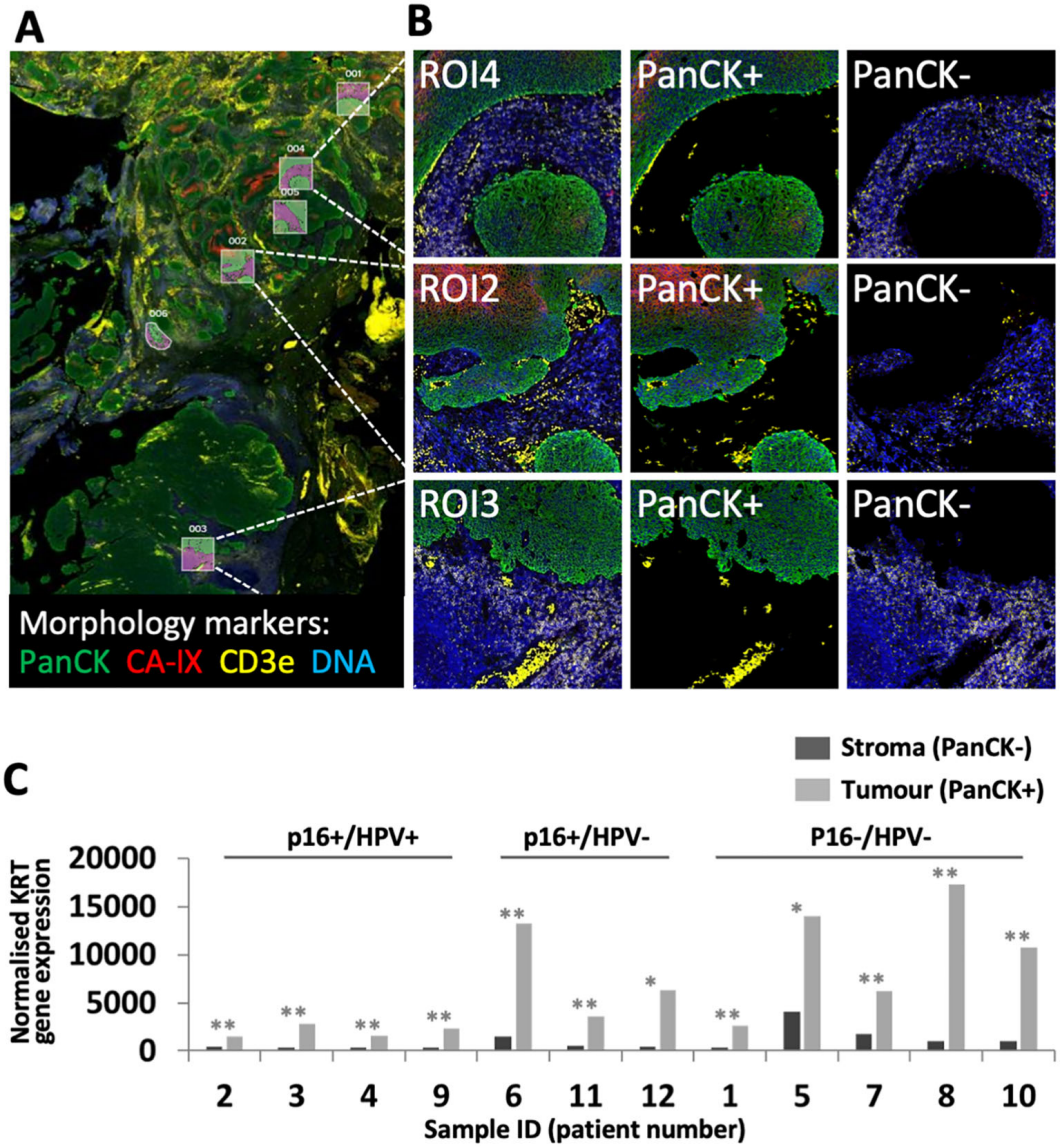
GeoMx DSP ROI selection and segmentation approach. **(A)** Tumour sections were stained with three morphology markers: PanCK (tumour; green), CD3e (T cell marker; yellow), CA-IX (surrogate marker for regions of hypoxia; red), plus a DNA stain to identify all cells (blue). ROIs were placed to identify regions within tumour nests, tumour-stroma interface and peri-tumoural stroma. In this illustrative example from patient 11, ROIs were placed to capture tumour and adjacent stroma in regions of high (ROIs 1, 2, 4, 5) and low (ROIs 3, 6) hypoxia. **(B)** Segmentation strategy – PanCK staining was used for identification of tumour (PanCK+) and stromal regions (PanCK-), enabling separate analysis. **(C)** Comparison of keratin gene expression between stromal and tumour compartments. Graph shows combined normalised KRT gene expression for all PanCK- versus PanCK+ segments within each of the 12 patient samples. P values for comparison of PanCK- versus PanCK+ were calculated using unpaired t test, adjusted p values reported as * <0.05, ** <0.005.

The cohort of 12 FFPE diagnostic biopsy samples from patients with OPC included four patients whose tumours were p16+/HPV+, three discordant (p16+/HPV-), and five p16-/HPV-. Patient characteristics are summarised in **Table 2**. Gene expression was analysed separately within the tumour (PanCK+) and stromal regions (PanCK-). ROIs were placed to capture gene expression within the tumour core, at the tumour-stroma interface and within the peritumoural stroma. Additional morphology markers were used to identify regions with high versus low T cell density (identified by CD3e) and high versus low hypoxia (as determined by CA-IX staining). Morphology marker staining, ROI placement and segmentation strategy is illustrated for one sample in **Figures 1A, B**. Comparison of keratin gene expression within the tumour and stromal compartments (**Figure 1C**), confirmed the efficacy of the segmentation approach.

**Table 2:** Patient characteristics.

Patient no.	Tumour subsite	Gender	Age group	Clinical stage (TNM7)	HPV status 0 = negative 1 = positive	p16 status 0 = negative 1 = positive	Smoking status 1 = never 2 = past 3 = current	Alcohol consumption 1 = low 2 = moderate 3 = heavy
**2**	Tonsil	M	66–70	IV	1	1	3	2
**3**	Tonsil	M	51–55	NA	1	1	NA	NA
**4**	Tonsil	M	66–70	IV	1	1	1	2
**9**	NA	M	46–50	IV	1	1	3	2
**6**	Posterior pharyngeal wall	M	56–60	IV	0	1	3	3
**11**	Tonsil	M	36–40	IV	0	1	1	2
**12**	Tonsil	M	56–60	IV	0	1	2	3
**1**	Tonsil	M	61–65	IV	0	0	3	2
**5**	Tonsil	M	61–65	IV	0	0	1	3
**7**	Soft palate	M	61–65	II	0	0	3	2
**8**	Tonsil	M	51–55	III	0	0	3	3
**10**	NA	M	66–70	IV	0	0	3	2

NA, not available.

**Figure 2:**
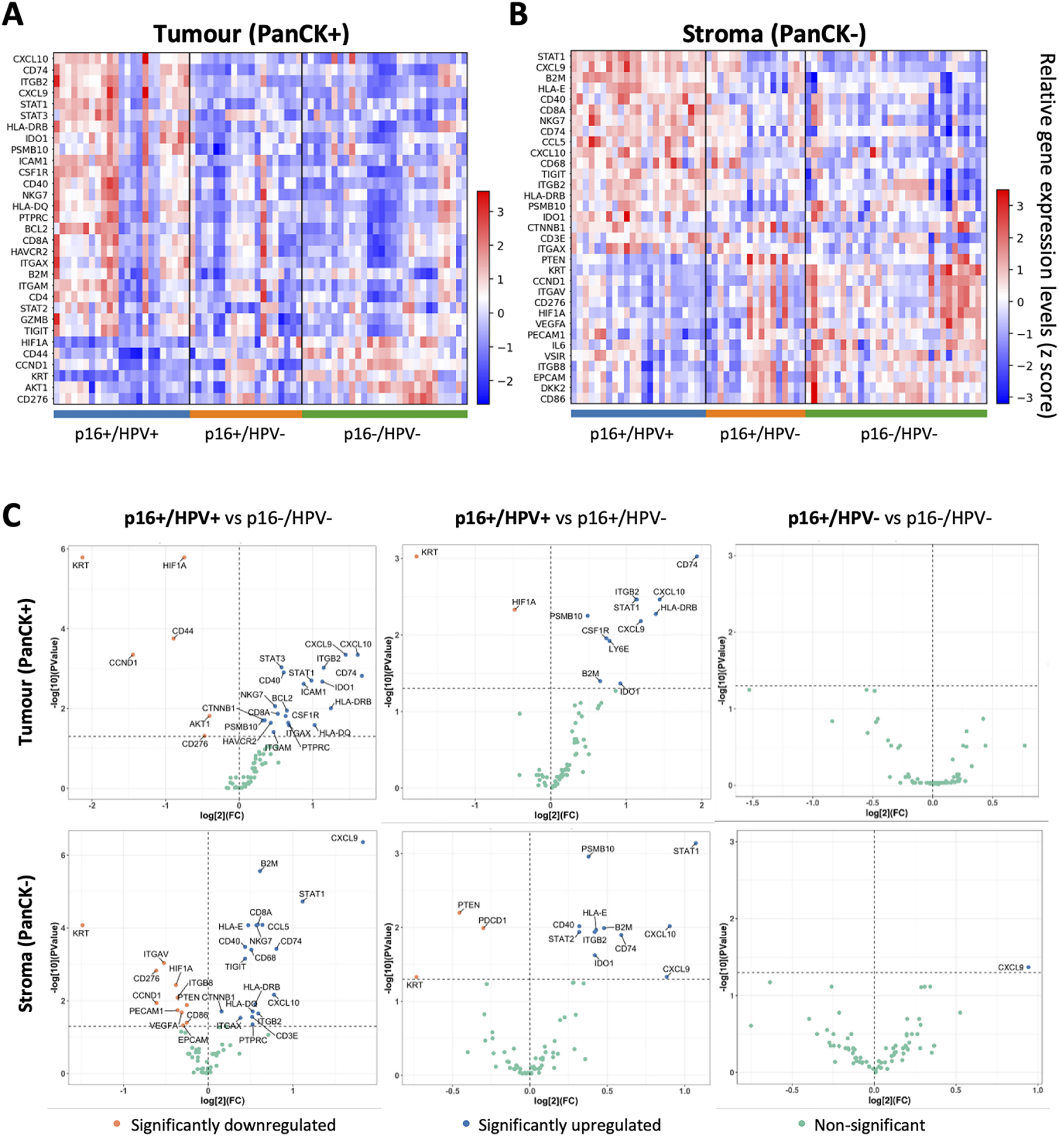
Identification of genes differentially expressed between p16+/HPV+, p16+/HPV- and p16-/HPV- oropharyngeal tumours. **(A, B)**. Heatmaps showing relative gene expression levels for significantly differentially expressed genes across p16/HPV subgroups within the tumour and stromal compartments respectively. Gene names are indicated for rows, ordered by the “scores” generated from the Wilcoxon rank-sum test statistics. Each column represents an individual ROI (p16+/HPV+: n = 46; p16+/HPV-: n = 36; p16-/HPV-: n = 59. **(C)** Volcano plots showing pairwise comparisons of gene expression between the three groups for the tumour (top) and stromal (bottom) compartments. The p16/HPV subgroup listed first is the primary group, while the subgroup listed second is the reference group. Significantly down regulated genes in the primary group are shown in pink, significantly upregulated genes in blue and non-significantly altered genes in green. P values for pair-wise comparison between any two groups were calculated using the Wilcoxon rank-sum test and adjusted by the Benjamini-Hochberg method across all genes.

Following data QC and normalisation, genes differentially expressed between the three p16/HPV subgroups were identified. Heatmaps illustrating significantly differentially expressed genes in each subgroup relative to the other two subgroups are shown in **Figures 2A, B**, for the tumour and stromal compartments respectively. Pairwise comparisons of expression of all immuno-oncology-related genes between the three different p16/HPV subgroups (p16+/HPV+ versus p16-/HPV-, p16+/HPV+ versus p16+/HPV- and p16+/HPV- versus p16-/HPV-) are illustrated in **Figure 2C** and listed in **Supplementary Table 1**. Genes whose expression is significantly down-regulated within the tumour compartment of p16+/HPV+ versus p16+/HPV- and/or p16-/HPV- tumours include keratins (KRT), *HIF1A*, *CD44*, *CCND1*, *AKT1* and *CD276* (**Figure 2C** top left and centre panels). The expression levels of *HIF1A*, *CCND1* and *CD276* are also downregulated in the stromal compartment when comparing the p16+/HPV+ group versus the p16-/HPV- group (**Figure 2C** bottom left panel). Other genes displaying reduced expression in the stromal compartment include *PTEN*, *ITGAV*, *ITGB8* and *PDCD1* (**Figure 2C** left and centre panels). Multiple genes are up-regulated within the tumour and stromal compartments of p16+/HPV+ tumours relative to p16+/HPV- and p16-/HPV- (**Figure 2C** left and centre panels), including several whose function relates to anti-tumoural immune responses (*CXCL9*, *CXCL10*, *CCL5*, *STAT1*, *ITGB2, PSMB10, B2M*, *CD74* and HLA-DRB). Of note, in these pairwise comparisons only one gene (*CXCL9*) showed significantly different expression between p16+/HPV- and p16-/HPV- tumours, being overexpressed in the stromal compartment of p16+/HPV- OPC (**Figure 2C** right hand panels). Three additional genes, *CCND1*, *AKT1* and *CD44* displayed a trend for reduced expression in the tumoural compartment of p16+/HPV- OPCs relative to p16-/HPV- (adjusted p values <0.06) see **Supplementary Table 1**.

**Figure 3:**
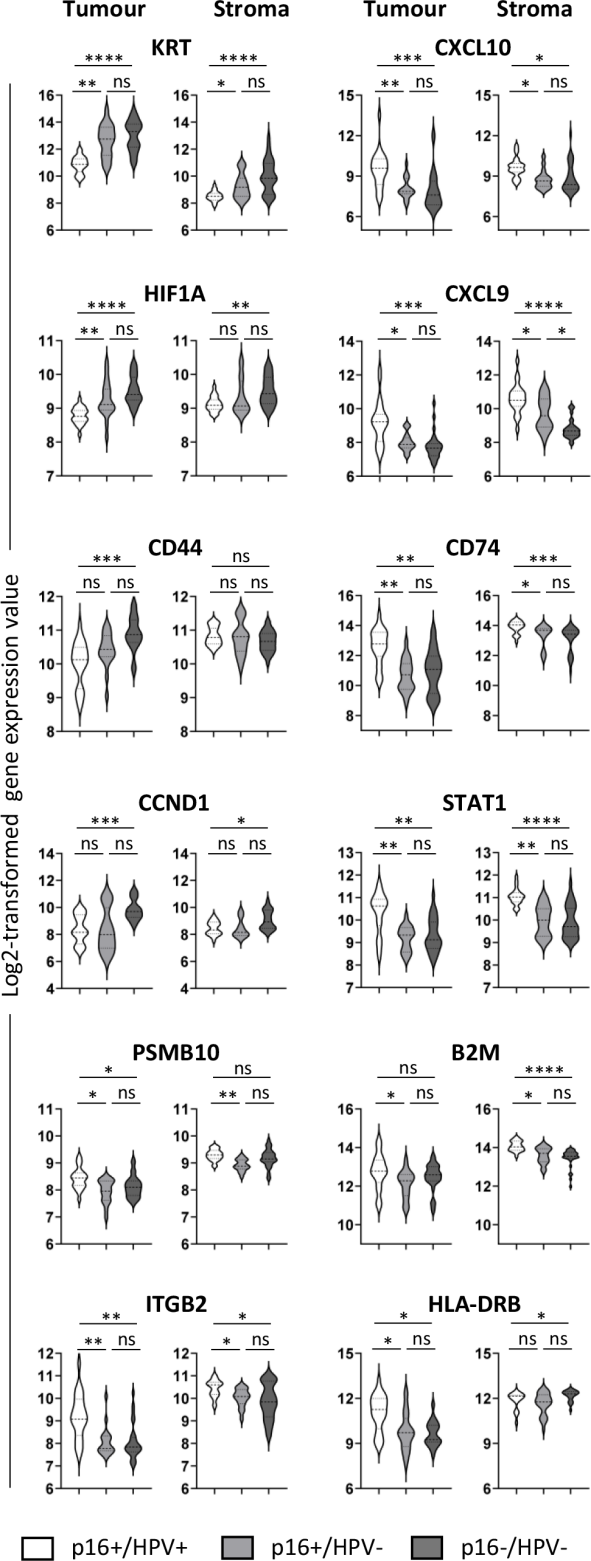
Expression of selected immuno-oncology-related genes within the three p16/HPV subgroups. Violin plots showing log2-transformed normalised gene expression for p16+/HPV+ (white), p16+/HPV- (pale grey) and p16-/HPV- (dark grey) OPC samples. First and third columns represent tumour (PanCK+), second and fourth columns represent stroma (PanCK-). Dashed and dotted lines represent the median and quartiles respectively. Data were analysed using Kruskal–Wallis test, and pair-wise comparisons were conducted with the Wilcoxon rank-sum test. Adjusted P values (see **Supplementary Table 1**) are reported as: ns, nonsignificant; *P < 0.05; **P < 0.005; ***P < 0.0005; ****P < 0.0001.

Differential expression of selected genes (those showing the most significant differences in the pairwise comparisons) between all three groups is presented in **Figure 3**. The violin plots illustrate both inter-group and within-group differences in gene expression. In respect of inter-group differences, **Figure 3** again highlights the similarity between the p16+/HPV- and p16-/HPV- groups and differential expression relative to the p16+/HPV+ group. With respect to the within-group distribution, some genes have a narrow expression range (for example, KRT, *HIF1A* in both tumour and stromal compartments of p16+/HPV+ tumours, and *PSMB10*, *B2M* in the stromal compartment for all p16/HPV subgroups). Conversely, many genes have broad, often bi-/multimodal distribution (for example *CD44*, *CCND1* and *CD74* in tumour and *STAT1* in both compartments for all p16/HPV subgroups), potentially reflecting spatial context – tumour core versus tumour periphery or tumour-adjacent versus more distant stroma.

## Discussion

Spatially resolved analysis of gene (or protein) expression within distinct tumour regions offers valuable insights for unravelling tumour composition and microenvironmental phenotypes (23, 24). Here, we employed GeoMx DSP to partition OPC tissues into tumour (PanCK+) and stromal (PanCK-) compartments and quantitatively assessed gene expression within each segmented region. This approach provides valuable information that is lost in bulk RNA-sequencing. Previous studies in HNC have successfully utilised this platform to analyse the tumour and microenvironment in patients with recurrent/metastatic HNC treated with immune checkpoint inhibitors (25–27). Here, we analysed expression of immuno-oncology-related genes in whole tissue sections from locally-advanced OPC patients treated with chemoradiotherapy +/- surgery in the curative setting, in relation to their p16 and HPV status. Overall, p16+/HPV- and p16-/HPV- tumours showed highly similar gene expression profiles – in both tumour and stromal compartments – which are quite distinct from the pattern of gene expression displayed by p16+/HPV+ tumours.

The ‘gene family’ displaying most consistent differential expression between the three groups was KRT, being overexpressed in both p16-/HPV- and p16+/HPV- tumours relative to p16+/HPV+ tumours. This is consistent with histologic classification of OPC, whereby HPV-related tumours are described as non-keratinising (28, 29). Elevated keratin expression, particularly KRT17, is associated with poor prognosis and decreased survival in multiple cancer types (30) including OPC (31), potentially due to inhibition of T cell infiltration (32). Previous studies in HNC link cytokeratin upregulation (including KRT17) with HPV-negative status (33) or an ‘immune low’ subset of HPV-positive tumours (34). In these studies, HPV status was assigned based on detection of HPV DNA/RNA/gene signature and the potential effects of discordant p16/HPV status were not explored. The differential expression of KRT (albeit at very low levels) within the stromal compartment of OPCs mirrors expression patterns in the tumoural compartment, probably reflecting imperfect tumour-stroma segmentation – especially where tumours are discontinuous.

*HIF1A* gene expression is upregulated in the tumour compartment of both p16+/HPV- and p16-/HPV- OPC relative to p16+/HPV+, and also in the stromal compartment of p16-/HPV-. HIF-1α comprises one subunit of the heterodimeric HIF1 protein, a key transcriptional regulator of cellular adaptation to low oxygen levels. While control of HIF-1α expression is mostly achieved through post-translational regulation (reduced degradation leading to protein accumulation under hypoxic conditions), transcriptional regulation also plays a role (35, 36). High tumoural hypoxia is associated with poor prognosis (37, 38), mediated by multiple factors including radiotherapy/chemotherapy resistance, heightened immunosuppressive nature of the tumour microenvironment, and increased epithelial-to-mesenchymal transition facilitating metastasis (39, 40). Although the HPV E7 oncogene has been shown to upregulate *HIF1A* expression (41), previous literature associates highest *HIF1A* expression with HPV-negative status (33). Here, we identified that this association extends to include p16+/HPV- discordant tumours and not just classical p16-/HPV-, a finding that could have important implications for future treatment strategies.

*CCND1*, *AKT1* and *CD44* were all expressed at significantly higher levels in the tumour compartment of p16-/HPV- relative to p16+/HPV+ OPC, with *CCND1* also being upregulated in the stromal compartment. *CCND1* (Cyclin D1) gene amplification, a relatively frequent event in head and neck cancer, has been associated with HPV-negative status and poor survival (33, 42). Mechanistically, this is linked to enhanced tumour cell proliferation and invasion, with recent evidence supporting a role for stromal expression in promoting inflammation and immunosuppression (43). CD44 has been widely implicated as a marker of cancer stem cells in multiple cancer types including OPC (44). High expression is associated with treatment resistance, epithelial-to-mesenchymal transition, and poor survival in most studies (45). Higher expression of *CCND1* and *CD44* in p16-/HPV- tumours relative to p16+/HPV+ aligns with their poorer prognosis. The trend for reduced expression of all three genes in discordant p16+/HPV- tumours compared with p16-/HPV- is consistent with intermediate prognosis (20).

Phenotypic and transcriptomic studies have characterised HPV-positive tumours as ‘immune hot’ (46–48), with greater numbers of peritumoural immune cells and increased intratumoural T cell infiltration relative to HPV-negative tumours. Many of the genes upregulated in p16+/HPV+ tumours (tumoural and stromal compartments) have potential roles in anti-tumoural immunity. These include, for example, key chemokines controlling T cell trafficking (*CXCL9*, *CXCL10*), adhesion molecules involved in T cell recruitment (*ITGB2*) and components of antigen processing and presentation pathways for CD4 or CD8 T cell recognition (*B2M*, *PBSM10*, *CD74*, HLA-DRB). STAT1, a key transcription factor shaping the tumour microenvironment, is also upregulated in both the tumour and stromal compartments of p16+/HPV+ tumours. Although STAT1 expression is essential for effective antitumor T cell responses (49, 50), tumour cell-specific expression may have negative consequences (50, 51). Of note, expression of anti-tumour immune response-related genes is down-regulated in both p16+/HPV- and HPV-/p16- tumours, consistent with the poorer survival outcomes of both these subgroups relative to p16+/HPV+. CXCL9 upregulation in the stromal compartment of p16+/HPV- tumours relative to p16-/HPV- is suggestive of subtle amelioration of immune responses consistent with intermediate prognosis.

Our study has several limitations. Firstly, the sample size is small, reflecting the capacity of the GeoMx DSP platform which provides in depth analysis rather than high throughput screening. Secondly, the cohort does not include any examples of the second discordant group (p16-/HPV+). This subgroup is much less clinically relevant as it is smaller and also not misdiagnosed (i.e. treated as p16+/HPV+, unlike the p16+/HPV- discordant subgroup) if p16 IHC is used on its own. Thirdly, we here present analysis of 73 immuno-oncology-related genes; GeoMx DSP technology has now progressed to enable whole transcriptome analysis, which we are currently employing in an extended cohort to provide further information on mechanistic differences. For example, this might further define an intermediate microenvironmental p16+/HPV- discordant phenotype, as hinted by differential CXCL9 expression, which aligns with the observed intermediate clinical outcome.

In summary, our study demonstrates that, in terms of immuno-oncology-related gene expression, p16+/HPV- OPC are much more closely aligned with p16-/HPV- than p16+/HPV+, although some subtle differences exist between p16+/HPV- and p16-/HPV- tumours. This is consistent with prognostic patterns described in a large pivotal study, p16+/HPV+ >> p16+/HPV- > p16-/HPV- and underlines the need for dual testing to support informed clinical decision making.

## Data Availability

The raw data supporting the conclusions of this article will be made available by the authors, without undue reservation.
