# Supplementary material for: Digital Spatial Profiling identifies distinct patterns of immuno-oncology-related gene expression within oropharyngeal tumours in relation to HPV and p16 status
Source: Front Oncol. 2024 Sep 12;14:1428741. doi: 10.3389/fonc.2024.1428741 (PMC11424609; doi:10.3389/fonc.2024.1428741)
Supplement: Supplementary file 1 [file DataSheet1.pdf]

Supplementary Table 1. Relative expression of immuno-oncology-related genes in p16+/HPV+, p16+/HPV- and p16-/HPV- OPC. Table shows results of pairwise comparisons of gene expression (n=73) between the three groups (p16+/HPV+ versus p16-/HPV-, p16+/HPV+ versus p16+/HPV-, p16-/HPV+ versus p16-/HPV-). Genes showing significantly different expression (adjusted p value <0.05) are emboldened.

Segment	Gene name	Group 1	Group 2	Mean group 1 (log2 count)	Mean group 2 (log2 count)	Log fold change (group 1 vs. group 2)	P value	Adjusted p-value
**PanCK+**	***CXCL10***	p16+/HPV+	p16-/HPV-	9.516	7.906	1.609	0.0000339	0.000447748
**PanCK+**	***CXCL9***	p16+/HPV+	p16-/HPV-	9.2	7.754	1.446	0.0000368	0.000447748
**PanCK+**	***STAT3***	p16+/HPV+	p16-/HPV-	9.781	9.207	0.574	0.0000891	0.000929325
**PanCK+**	***ITGB2***	p16+/HPV+	p16-/HPV-	9.202	8.056	1.146	0.000104195	0.000950782
**PanCK+**	***CD40***	p16+/HPV+	p16-/HPV-	7.723	7.119	0.604	0.000153116	0.001241944
**PanCK+**	***CD74***	p16+/HPV+	p16-/HPV-	12.602	10.936	1.666	0.000207062	0.001511554
**PanCK+**	***STAT1***	p16+/HPV+	p16-/HPV-	10.318	9.339	0.979	0.000299662	0.001988668
**PanCK+**	***IDO1***	p16+/HPV+	p16-/HPV-	8.7	7.573	1.127	0.000346588	0.002108413
**PanCK+**	***ICAM1***	p16+/HPV+	p16-/HPV-	8.996	8.121	0.875	0.000430015	0.002414702
**PanCK+**	***NKG7***	p16+/HPV+	p16-/HPV-	7.845	7.359	0.486	0.001675994	0.008739113
**PanCK+**	**HLA-DRB**	p16+/HPV+	p16-/HPV-	11.02	9.775	1.244	0.00203171	0.009887657
**PanCK+**	***BCL2***	p16+/HPV+	p16-/HPV-	7.969	7.323	0.646	0.002455532	0.011203366
**PanCK+**	***CD8A***	p16+/HPV+	p16-/HPV-	8.161	7.637	0.524	0.003146523	0.013511539
**PanCK+**	***CSF1R***	p16+/HPV+	p16-/HPV-	7.207	6.577	0.629	0.004010565	0.015409014
**PanCK+**	***CTNNB1***	p16+/HPV+	p16-/HPV-	9.967	9.623	0.345	0.005391186	0.019677829
**PanCK+**	***PSMB10***	p16+/HPV+	p16-/HPV-	8.437	8.12	0.317	0.005714117	0.019863359
**PanCK+**	***PTPRC***	p16+/HPV+	p16-/HPV-	8.258	7.593	0.665	0.007187461	0.022812375
**PanCK+**	***HAVCR2***	p16+/HPV+	p16-/HPV-	7.999	7.572	0.427	0.007187461	0.022812375
**PanCK+**	**HLA-DQ**	p16+/HPV+	p16-/HPV-	9.08	8.058	1.022	0.00850734	0.025876491
**PanCK+**	***ITGAX***	p16+/HPV+	p16-/HPV-	8.153	7.477	0.676	0.008993205	0.026260158
**PanCK+**	***ITGAM***	p16+/HPV+	p16-/HPV-	7.665	7.199	0.467	0.013859905	0.038914348
**PanCK+**	*EPCAM*	p16+/HPV+	p16-/HPV-	7.541	7.029	0.512	0.037317088	0.090804913
**PanCK+**	*TIGIT*	p16+/HPV+	p16-/HPV-	7.805	7.427	0.378	0.037317088	0.090804913
**PanCK+**	*ICOSLG*	p16+/HPV+	p16-/HPV-	7.782	7.361	0.422	0.037317088	0.090804913
**PanCK+**	*CD4*	p16+/HPV+	p16-/HPV-	8.549	8.2	0.349	0.044796666	0.105488923
**PanCK+**	*IFNAR1*	p16+/HPV+	p16-/HPV-	7.847	7.578	0.269	0.053502896	0.122053481
**PanCK+**	pan-melanocyte	p16+/HPV+	p16-/HPV-	8.719	8.39	0.329	0.06357904	0.138344339
**PanCK+**	*CD3E*	p16+/HPV+	p16-/HPV-	7.744	7.318	0.426	0.066329477	0.138344339
**PanCK+**	*TNFRSF9*	p16+/HPV+	p16-/HPV-	7.458	7.18	0.279	0.066329477	0.138344339
**PanCK+**	*TBX21*	p16+/HPV+	p16-/HPV-	7.678	7.456	0.222	0.075174015	0.152436198
**PanCK+**	*TNF*	p16+/HPV+	p16-/HPV-	7.721	7.501	0.22	0.092036397	0.181585323
**PanCK+**	*LAG3*	p16+/HPV+	p16-/HPV-	7.525	7.244	0.281	0.099580129	0.19129867
**PanCK+**	*MS4A1*	p16+/HPV+	p16-/HPV-	7.942	7.411	0.531	0.120603954	0.225745863
**PanCK+**	*GZMB*	p16+/HPV+	p16-/HPV-	7.862	7.656	0.206	0.125198563	0.228487377
**PanCK+**	*VEGFA*	p16+/HPV+	p16-/HPV-	9.181	8.826	0.355	0.129928482	0.231336078
**PanCK+**	*STAT2*	p16+/HPV+	p16-/HPV-	8.887	8.693	0.194	0.134795952	0.234288201
**PanCK+**	*FAS*	p16+/HPV+	p16-/HPV-	7.846	7.527	0.319	0.144952326	0.246081856
**PanCK+**	*B2M*	p16+/HPV+	p16-/HPV-	12.759	12.451	0.308	0.150245525	0.249270984
**PanCK+**	*FOXP3*	p16+/HPV+	p16-/HPV-	6.736	6.526	0.21	0.191499453	0.310654667
**PanCK+**	*CD274*	p16+/HPV+	p16-/HPV-	7.331	7.152	0.179	0.198015396	0.314241825
**PanCK+**	*IL15*	p16+/HPV+	p16-/HPV-	7.575	7.391	0.184	0.204692059	0.317925964

**PanCK+**	*IFNG*	p16+/HPV+	p16-/HPV-	5.348	5.116	0.231	0.256045309	0.381455257
**PanCK+**	*CD27*	p16+/HPV+	p16-/HPV-	7.265	7.058	0.207	0.256045309	0.381455257
**PanCK+**	*VSIR*	p16+/HPV+	p16-/HPV-	7.683	7.567	0.117	0.363545263	0.52036871
**PanCK+**	*CD40LG*	p16+/HPV+	p16-/HPV-	7.061	7.005	0.057	0.363545263	0.52036871
**PanCK+**	*PDCD1LG2*	p16+/HPV+	p16-/HPV-	7.102	6.944	0.157	0.383879321	0.53303837
**PanCK+**	*CD68*	p16+/HPV+	p16-/HPV-	9.159	8.955	0.205	0.394302356	0.53303837
**PanCK+**	*BATF3*	p16+/HPV+	p16-/HPV-	7.555	7.404	0.151	0.415655615	0.541836784
**PanCK+**	*CMKLR1*	p16+/HPV+	p16-/HPV-	7.607	7.451	0.156	0.532178878	0.681562422
**PanCK+**	*CTLA4*	p16+/HPV+	p16-/HPV-	7.254	7.127	0.127	0.557322058	0.701457073
**PanCK+**	*CXCR6*	p16+/HPV+	p16-/HPV-	7.332	7.229	0.104	0.636038403	0.761160711
**PanCK+**	*ITGB8*	p16+/HPV+	p16-/HPV-	8.768	8.64	0.128	0.649601983	0.764853948
**PanCK+**	*IL6*	p16+/HPV+	p16-/HPV-	7.097	7.038	0.06	0.663282712	0.768565682
**PanCK+**	*CCL5*	p16+/HPV+	p16-/HPV-	8.418	8.336	0.082	0.790996215	0.902230058
**PanCK+**	*IFNGR1*	p16+/HPV+	p16-/HPV-	8.133	8.113	0.02	0.909571695	0.967972861
**PanCK+**	*PECAM1*	p16+/HPV+	p16-/HPV-	7.401	7.399	0.002	0.954712959	0.967972861
**PanCK+**	*PDCD1*	p16+/HPV+	p16-/HPV-	7.669	7.632	0.036	0.954712959	0.967972861
**PanCK+**	***HIF1A***	p16+/HPV+	p16-/HPV-	8.782	9.53	-0.748	2.92E-08	0.00000164
**PanCK+**	**KRT**	p16+/HPV+	p16-/HPV-	10.862	12.994	-2.132	4.48E-08	0.00000164
**PanCK+**	***CD44***	p16+/HPV+	p16-/HPV-	10.003	10.896	-0.893	0.00000724	0.000176294
**PanCK+**	***CCND1***	p16+/HPV+	p16-/HPV-	8.401	9.846	-1.444	0.0000312	0.000447748
**PanCK+**	***AKT1***	p16+/HPV+	p16-/HPV-	9.32	9.725	-0.405	0.003776402	0.015315407
**PanCK+**	***CD276***	p16+/HPV+	p16-/HPV-	7.957	8.43	-0.473	0.017970521	0.048586965
**PanCK+**	*HLA-E*	p16+/HPV+	p16-/HPV-	10.497	10.541	-0.044	0.394302356	0.53303837
**PanCK+**	*CD86*	p16+/HPV+	p16-/HPV-	7.555	7.674	-0.119	0.415655615	0.541836784
**PanCK+**	*ARG1*	p16+/HPV+	p16-/HPV-	7.234	7.354	-0.12	0.583030025	0.721376133
**PanCK+**	*MKI67*	p16+/HPV+	p16-/HPV-	7.931	8.023	-0.093	0.636038403	0.761160711
**PanCK+**	*DKK2*	p16+/HPV+	p16-/HPV-	6.966	7.013	-0.047	0.820301199	0.921261347
**PanCK+**	*ITGAV*	p16+/HPV+	p16-/HPV-	9.157	9.215	-0.058	0.864720582	0.956433371
**PanCK+**	*IL12B*	p16+/HPV+	p16-/HPV-	7.009	7.024	-0.015	0.909571695	0.967972861
**PanCK+**	*LY6E*	p16+/HPV+	p16-/HPV-	9.691	9.749	-0.058	0.954712959	0.967972861
**PanCK+**	*PTEN*	p16+/HPV+	p16-/HPV-	8.543	8.693	-0.15	0.984897107	0.984897107
**PanCK+**	*CD47*	p16+/HPV+	p16-/HPV-	9.083	9.255	-0.171	0.954712959	0.967972861
**PanCK+**	***CD74***	p16+/HPV+	p16+/HPV-	12.602	10.667	1.935	0.0000258	0.000942238
**PanCK+**	***ITGB2***	p16+/HPV+	p16+/HPV-	9.202	8.061	1.141	0.000158121	0.003447209
**PanCK+**	***STAT1***	p16+/HPV+	p16+/HPV-	10.318	9.185	1.133	0.000193454	0.003447209
**PanCK+**	***CXCL10***	p16+/HPV+	p16+/HPV-	9.516	8.074	1.442	0.00023611	0.003447209
**PanCK+**	**HLA-DRB**	p16+/HPV+	p16+/HPV-	11.02	9.629	1.391	0.000511409	0.005333263
**PanCK+**	***PSMB10***	p16+/HPV+	p16+/HPV-	8.437	7.948	0.489	0.000616686	0.005627264
**PanCK+**	***CXCL9***	p16+/HPV+	p16+/HPV-	9.2	8.008	1.192	0.000812927	0.006593739
**PanCK+**	***CSF1R***	p16+/HPV+	p16+/HPV-	7.207	6.47	0.737	0.001516741	0.011072211
**PanCK+**	***LY6E***	p16+/HPV+	p16+/HPV-	9.691	8.912	0.779	0.00180287	0.011964502
**PanCK+**	***B2M***	p16+/HPV+	p16+/HPV-	12.759	12.104	0.655	0.006595821	0.040124575
**PanCK+**	***IDO1***	p16+/HPV+	p16+/HPV-	8.7	7.778	0.922	0.007674849	0.043097231
**PanCK+**	HLA-DQ	p16+/HPV+	p16+/HPV-	9.08	8.221	0.859	0.010318573	0.053803987
**PanCK+**	*ICAM1*	p16+/HPV+	p16+/HPV-	8.996	8.326	0.671	0.016937006	0.077275089
**PanCK+**	*STAT3*	p16+/HPV+	p16+/HPV-	9.781	9.383	0.398	0.016937006	0.077275089
**PanCK+**	*STAT2*	p16+/HPV+	p16+/HPV-	8.887	8.567	0.32	0.019411582	0.083355619
**PanCK+**	*PTPRC*	p16+/HPV+	p16+/HPV-	8.258	7.62	0.638	0.020763329	0.084206834
**PanCK+**	*CCL5*	p16+/HPV+	p16+/HPV-	8.418	7.793	0.625	0.023714749	0.091114562

**PanCK+**	*NKG7*	p16+/HPV+	p16+/HPV-	7.845	7.471	0.374	0.028822372	0.105201658
**PanCK+**	*GZMB*	p16+/HPV+	p16+/HPV-	7.862	7.451	0.411	0.034850114	0.115639014
**PanCK+**	*HAVCR2*	p16+/HPV+	p16+/HPV-	7.999	7.669	0.329	0.044535726	0.141352523
**PanCK+**	*CD47*	p16+/HPV+	p16+/HPV-	9.083	8.799	0.285	0.059746428	0.181728718
**PanCK+**	*CD8A*	p16+/HPV+	p16+/HPV-	8.161	7.82	0.34	0.083445238	0.229563253
**PanCK+**	*BATF3*	p16+/HPV+	p16+/HPV-	7.555	7.233	0.322	0.088051659	0.229563253
**PanCK+**	*CD4*	p16+/HPV+	p16+/HPV-	8.549	8.231	0.318	0.088051659	0.229563253
**PanCK+**	*CD40*	p16+/HPV+	p16+/HPV-	7.723	7.401	0.322	0.088051659	0.229563253
**PanCK+**	*BCL2*	p16+/HPV+	p16+/HPV-	7.969	7.53	0.438	0.092860973	0.233753484
**PanCK+**	*ITGB8*	p16+/HPV+	p16+/HPV-	8.768	8.414	0.354	0.103111185	0.250903884
**PanCK+**	*ITGAV*	p16+/HPV+	p16+/HPV-	9.157	8.843	0.314	0.108563463	0.255649444
**PanCK+**	*ITGAX*	p16+/HPV+	p16+/HPV-	8.153	7.694	0.459	0.126296245	0.288113308
**PanCK+**	*CD3E*	p16+/HPV+	p16+/HPV-	7.744	7.402	0.342	0.160760411	0.355621515
**PanCK+**	*MS4A1*	p16+/HPV+	p16+/HPV-	7.942	7.437	0.505	0.168435558	0.361641051
**PanCK+**	*TIGIT*	p16+/HPV+	p16+/HPV-	7.805	7.476	0.328	0.176382518	0.367883538
**PanCK+**	*FAS*	p16+/HPV+	p16+/HPV-	7.846	7.629	0.218	0.250221054	0.493679376
**PanCK+**	*IFNGR1*	p16+/HPV+	p16+/HPV-	8.133	7.916	0.218	0.250221054	0.493679376
**PanCK+**	*ITGAM*	p16+/HPV+	p16+/HPV-	7.665	7.417	0.249	0.260783155	0.500978167
**PanCK+**	*VSIR*	p16+/HPV+	p16+/HPV-	7.683	7.47	0.214	0.294302312	0.550873558
**PanCK+**	*AKT1*	p16+/HPV+	p16+/HPV-	9.32	9.174	0.146	0.330593871	0.578049491
**PanCK+**	*TNF*	p16+/HPV+	p16+/HPV-	7.721	7.572	0.15	0.343308799	0.578049491
**PanCK+**	*ICOSLG*	p16+/HPV+	p16+/HPV-	7.782	7.587	0.196	0.343308799	0.578049491
**PanCK+**	*LAG3*	p16+/HPV+	p16+/HPV-	7.525	7.292	0.233	0.356331878	0.578049491
**PanCK+**	*TNFRSF9*	p16+/HPV+	p16+/HPV-	7.458	7.272	0.186	0.356331878	0.578049491
**PanCK+**	*CD68*	p16+/HPV+	p16+/HPV-	9.159	9.001	0.158	0.383297881	0.608277072
**PanCK+**	*HLA-E*	p16+/HPV+	p16+/HPV-	10.497	10.069	0.428	0.39723744	0.616985811
**PanCK+**	*CMKLR1*	p16+/HPV+	p16+/HPV-	7.607	7.4	0.207	0.440853043	0.670464003
**PanCK+**	*IL15*	p16+/HPV+	p16+/HPV-	7.575	7.359	0.215	0.471394289	0.676965202
**PanCK+**	*PECAM1*	p16+/HPV+	p16+/HPV-	7.401	7.237	0.164	0.48709184	0.676965202
**PanCK+**	*FOXP3*	p16+/HPV+	p16+/HPV-	6.736	6.595	0.14	0.503067485	0.676965202
**PanCK+**	*CD27*	p16+/HPV+	p16+/HPV-	7.265	7.051	0.213	0.519315772	0.676965202
**PanCK+**	*IFNG*	p16+/HPV+	p16+/HPV-	5.348	5.238	0.108	0.569635321	0.71407485
**PanCK+**	*CCND1*	p16+/HPV+	p16+/HPV-	8.401	8.315	0.085	0.586910836	0.71407485
**PanCK+**	*CTLA4*	p16+/HPV+	p16+/HPV-	7.254	7.106	0.148	0.586910836	0.71407485
**PanCK+**	*PDCD1LG2*	p16+/HPV+	p16+/HPV-	7.102	6.985	0.116	0.658318709	0.775117189
**PanCK+**	*EPCAM*	p16+/HPV+	p16+/HPV-	7.541	7.469	0.072	0.771348095	0.853157741
**PanCK+**	*CD40LG*	p16+/HPV+	p16+/HPV-	7.061	6.968	0.091	0.810275395	0.882837371
**PanCK+**	pan-melanocyte	p16+/HPV+	p16+/HPV-	8.719	8.662	0.056	0.849677794	0.912154103
**PanCK+**	*CXCR6*	p16+/HPV+	p16+/HPV-	7.332	7.305	0.027	0.929521308	0.942431326
**PanCK+**	*MKI67*	p16+/HPV+	p16+/HPV-	7.931	7.871	0.059	0.929521308	0.942431326
**PanCK+**	*TBX21*	p16+/HPV+	p16+/HPV-	7.678	7.66	0.019	0.989918755	0.989918755
**PanCK+**	*CTNNB1*	p16+/HPV+	p16+/HPV-	9.967	9.906	0.062	0.71404886	0.814461981
**PanCK+**	**KRT**	p16+/HPV+	p16+/HPV-	10.862	12.638	-1.775	0.0000184	0.000942238
**PanCK+**	***HIF1A***	p16+/HPV+	p16+/HPV-	8.782	9.258	-0.477	0.00038447	0.004677724
**PanCK+**	*CD44*	p16+/HPV+	p16+/HPV-	10.003	10.413	-0.41	0.030723398	0.106800382
**PanCK+**	*IFNAR1*	p16+/HPV+	p16+/HPV-	7.847	7.999	-0.152	0.343308799	0.578049491
**PanCK+**	*CD276*	p16+/HPV+	p16+/HPV-	7.957	8.142	-0.186	0.455979838	0.676965202
**PanCK+**	*VEGFA*	p16+/HPV+	p16+/HPV-	9.181	9.593	-0.412	0.503067485	0.676965202
**PanCK+**	*CD86*	p16+/HPV+	p16+/HPV-	7.555	7.716	-0.161	0.519315772	0.676965202

**PanCK+**	*PDCD1*	p16+/HPV+	p16+/HPV-	7.669	7.785	-0.117	0.519315772	0.676965202
**PanCK+**	*ARG1*	p16+/HPV+	p16+/HPV-	7.234	7.335	-0.102	0.569635321	0.71407485
**PanCK+**	*IL6*	p16+/HPV+	p16+/HPV-	7.097	7.147	-0.051	0.604425166	0.723328477
**PanCK+**	*CD274*	p16+/HPV+	p16+/HPV-	7.331	7.477	-0.147	0.676703995	0.784117328
**PanCK+**	*DKK2*	p16+/HPV+	p16+/HPV-	6.966	7.046	-0.08	0.771348095	0.853157741
**PanCK+**	*PTEN*	p16+/HPV+	p16+/HPV-	8.543	8.705	-0.162	0.869527299	0.919934678
**PanCK+**	*IL12B*	p16+/HPV+	p16+/HPV-	7.009	7.075	-0.067	0.889459363	0.92757905
**PanCK+**	*IFNAR1*	p16+/HPV-	p16-/HPV-	7.999	7.578	0.421	0.00928631	0.135580129
**PanCK+**	*CTNNB1*	p16+/HPV-	p16-/HPV-	9.906	9.623	0.283	0.021570816	0.1968337
**PanCK+**	*VEGFA*	p16+/HPV-	p16-/HPV-	9.593	8.826	0.767	0.051057351	0.303103255
**PanCK+**	*CXCL9*	p16+/HPV-	p16-/HPV-	8.008	7.754	0.254	0.059299781	0.303103255
**PanCK+**	*EPCAM*	p16+/HPV-	p16-/HPV-	7.469	7.029	0.44	0.062281491	0.303103255
**PanCK+**	*CD40*	p16+/HPV-	p16-/HPV-	7.401	7.119	0.282	0.068617356	0.313066686
**PanCK+**	*CXCL10*	p16+/HPV-	p16-/HPV-	8.074	7.906	0.167	0.113541017	0.460471901
**PanCK+**	*CD274*	p16+/HPV-	p16-/HPV-	7.477	7.152	0.325	0.14638786	0.562437567
**PanCK+**	*ITGAX*	p16+/HPV-	p16-/HPV-	7.694	7.477	0.217	0.208641601	0.718703228
**PanCK+**	*ICAM1*	p16+/HPV-	p16-/HPV-	8.326	8.121	0.205	0.216595493	0.718703228
**PanCK+**	*BCL2*	p16+/HPV-	p16-/HPV-	7.53	7.323	0.207	0.268914754	0.779046514
**PanCK+**	*STAT3*	p16+/HPV-	p16-/HPV-	9.383	9.207	0.176	0.268914754	0.779046514
**PanCK+**	*ICOSLG*	p16+/HPV-	p16-/HPV-	7.587	7.361	0.226	0.329312938	0.828960155
**PanCK+**	HLA-DQ	p16+/HPV-	p16-/HPV-	8.221	8.058	0.163	0.362577085	0.880985611
**PanCK+**	*TBX21*	p16+/HPV-	p16-/HPV-	7.66	7.456	0.203	0.422508579	0.88123218
**PanCK+**	pan-melanocyte	p16+/HPV-	p16-/HPV-	8.662	8.39	0.272	0.422508579	0.88123218
**PanCK+**	*IL6*	p16+/HPV-	p16-/HPV-	7.147	7.038	0.11	0.422508579	0.88123218
**PanCK+**	*IDO1*	p16+/HPV-	p16-/HPV-	7.778	7.573	0.205	0.435156072	0.882399814
**PanCK+**	*ITGAM*	p16+/HPV-	p16-/HPV-	7.417	7.199	0.218	0.448019338	0.883930046
**PanCK+**	*CD8A*	p16+/HPV-	p16-/HPV-	7.82	7.637	0.183	0.474383086	0.911314875
**PanCK+**	*FAS*	p16+/HPV-	p16-/HPV-	7.629	7.527	0.101	0.515477483	0.925725602
**PanCK+**	*PDCD1*	p16+/HPV-	p16-/HPV-	7.785	7.632	0.153	0.558346533	0.925725602
**PanCK+**	*IFNG*	p16+/HPV-	p16-/HPV-	5.238	5.116	0.124	0.618069497	0.925725602
**PanCK+**	*TNF*	p16+/HPV-	p16-/HPV-	7.572	7.501	0.071	0.648941813	0.925725602
**PanCK+**	*CXCR6*	p16+/HPV-	p16-/HPV-	7.305	7.229	0.077	0.696389041	0.925725602
**PanCK+**	*NKG7*	p16+/HPV-	p16-/HPV-	7.471	7.359	0.112	0.696389041	0.925725602
**PanCK+**	*TNFRSF9*	p16+/HPV-	p16-/HPV-	7.272	7.18	0.092	0.745055259	0.925725602
**PanCK+**	*CD3E*	p16+/HPV-	p16-/HPV-	7.402	7.318	0.084	0.761517618	0.925725602
**PanCK+**	*ITGB2*	p16+/HPV-	p16-/HPV-	8.061	8.056	0.005	0.778088635	0.925725602
**PanCK+**	*CD86*	p16+/HPV-	p16-/HPV-	7.716	7.674	0.042	0.778088635	0.925725602
**PanCK+**	*FOXP3*	p16+/HPV-	p16-/HPV-	6.595	6.526	0.07	0.811528026	0.925725602
**PanCK+**	*MS4A1*	p16+/HPV-	p16-/HPV-	7.437	7.411	0.026	0.828381753	0.925725602
**PanCK+**	*HAVCR2*	p16+/HPV-	p16-/HPV-	7.669	7.572	0.098	0.828381753	0.925725602
**PanCK+**	*IL12B*	p16+/HPV-	p16-/HPV-	7.075	7.024	0.052	0.845314864	0.925725602
**PanCK+**	*LAG3*	p16+/HPV-	p16-/HPV-	7.292	7.244	0.048	0.845314864	0.925725602
**PanCK+**	*PDCD1LG2*	p16+/HPV-	p16-/HPV-	6.985	6.944	0.041	0.896513818	0.948485633
**PanCK+**	*DKK2*	p16+/HPV-	p16-/HPV-	7.046	7.013	0.034	0.948147528	0.961316244
**PanCK+**	*TIGIT*	p16+/HPV-	p16-/HPV-	7.476	7.427	0.05	0.965418151	0.965418151
**PanCK+**	*PTEN*	p16+/HPV-	p16-/HPV-	8.705	8.693	0.012	0.930901235	0.957123805
**PanCK+**	*PTPRC*	p16+/HPV-	p16-/HPV-	7.62	7.593	0.026	0.862319738	0.925725602
**PanCK+**	*CD68*	p16+/HPV-	p16-/HPV-	9.001	8.955	0.046	0.828381753	0.925725602
**PanCK+**	*CD4*	p16+/HPV-	p16-/HPV-	8.231	8.2	0.031	0.811528026	0.925725602

**PanCK+**	*CCND1*	p16+/HPV-	p16-/HPV-	8.315	9.846	-1.529	0.000910888	0.056614735
**PanCK+**	*AKT1*	p16+/HPV-	p16-/HPV-	9.174	9.725	-0.552	0.001551089	0.056614735
**PanCK+**	*CD44*	p16+/HPV-	p16-/HPV-	10.413	10.896	-0.483	0.002406275	0.058552693
**PanCK+**	*CD47*	p16+/HPV-	p16-/HPV-	8.799	9.255	-0.456	0.00817669	0.135580129
**PanCK+**	*LY6E*	p16+/HPV-	p16-/HPV-	8.912	9.749	-0.837	0.011916111	0.144979351
**PanCK+**	*HLA-E*	p16+/HPV-	p16-/HPV-	10.069	10.541	-0.472	0.014302068	0.149150143
**PanCK+**	*CCL5*	p16+/HPV-	p16-/HPV-	7.793	8.336	-0.542	0.025561597	0.207332955
**PanCK+**	*ITGAV*	p16+/HPV-	p16-/HPV-	8.843	9.215	-0.372	0.035488357	0.259065005
**PanCK+**	*HIF1A*	p16+/HPV-	p16-/HPV-	9.258	9.53	-0.272	0.053691188	0.303103255
**PanCK+**	*B2M*	p16+/HPV-	p16-/HPV-	12.104	12.451	-0.347	0.062281491	0.303103255
**PanCK+**	*CD276*	p16+/HPV-	p16-/HPV-	8.142	8.43	-0.287	0.075473611	0.324092564
**PanCK+**	*GZMB*	p16+/HPV-	p16-/HPV-	7.451	7.656	-0.205	0.208641601	0.718703228
**PanCK+**	KRT	p16+/HPV-	p16-/HPV-	12.638	12.994	-0.356	0.278414699	0.779046514
**PanCK+**	*IFNGR1*	p16+/HPV-	p16-/HPV-	7.916	8.113	-0.197	0.278414699	0.779046514
**PanCK+**	*ITGB8*	p16+/HPV-	p16-/HPV-	8.414	8.64	-0.226	0.288140492	0.779046514
**PanCK+**	*STAT2*	p16+/HPV-	p16-/HPV-	8.567	8.693	-0.126	0.329312938	0.828960155
**PanCK+**	*BATF3*	p16+/HPV-	p16-/HPV-	7.233	7.404	-0.171	0.374117177	0.880985611
**PanCK+**	*PSMB10*	p16+/HPV-	p16-/HPV-	7.948	8.12	-0.172	0.410079082	0.88123218
**PanCK+**	*CSF1R*	p16+/HPV-	p16-/HPV-	6.47	6.577	-0.108	0.515477483	0.925725602
**PanCK+**	*PECAM1*	p16+/HPV-	p16-/HPV-	7.237	7.399	-0.163	0.529575123	0.925725602
**PanCK+**	*CD40LG*	p16+/HPV-	p16-/HPV-	6.968	7.005	-0.035	0.558346533	0.925725602
**PanCK+**	*CD74*	p16+/HPV-	p16-/HPV-	10.667	10.936	-0.269	0.558346533	0.925725602
**PanCK+**	*STAT1*	p16+/HPV-	p16-/HPV-	9.185	9.339	-0.154	0.602878203	0.925725602
**PanCK+**	*MKI67*	p16+/HPV-	p16-/HPV-	7.871	8.023	-0.152	0.71248424	0.925725602
**PanCK+**	*VSIR*	p16+/HPV-	p16-/HPV-	7.47	7.567	-0.097	0.71248424	0.925725602
**PanCK+**	*CMKLR1*	p16+/HPV-	p16-/HPV-	7.4	7.451	-0.051	0.745055259	0.925725602
**PanCK+**	*CTLA4*	p16+/HPV-	p16-/HPV-	7.106	7.127	-0.021	0.811528026	0.925725602
**PanCK+**	HLA-DRB	p16+/HPV-	p16-/HPV-	9.629	9.775	-0.147	0.811528026	0.925725602
**PanCK+**	*IL15*	p16+/HPV-	p16-/HPV-	7.359	7.391	-0.031	0.862319738	0.925725602
**PanCK+**	*ARG1*	p16+/HPV-	p16-/HPV-	7.335	7.354	-0.018	0.930901235	0.957123805
**PanCK+**	*CD27*	p16+/HPV-	p16-/HPV-	7.051	7.058	-0.007	0.728708529	0.925725602
**PanCK-**	***CXCL9***	p16+/HPV+	p16-/HPV-	10.613	8.787	1.826	6.01E-09	0.000000439
**PanCK-**	***B2M***	p16+/HPV+	p16-/HPV-	14.073	13.462	0.611	7.49E-08	0.00000273
**PanCK-**	***STAT1***	p16+/HPV+	p16-/HPV-	11.046	9.933	1.113	0.000000774	0.0000188
**PanCK-**	***CD8A***	p16+/HPV+	p16-/HPV-	9.358	8.773	0.585	0.00000439	0.0000802
**PanCK-**	***CCL5***	p16+/HPV+	p16-/HPV-	9.746	9.107	0.639	0.00000564	0.0000824
**PanCK-**	***HLA-E***	p16+/HPV+	p16-/HPV-	11.918	11.447	0.471	0.00000851	0.0000842
**PanCK-**	***NKG7***	p16+/HPV+	p16-/HPV-	9.149	8.58	0.569	0.00000923	0.0000842
**PanCK-**	***CD40***	p16+/HPV+	p16-/HPV-	8.661	8.225	0.435	0.0000409	0.000332068
**PanCK-**	***CD74***	p16+/HPV+	p16-/HPV-	13.894	13.089	0.804	0.0000513	0.000374478
**PanCK-**	***CD68***	p16+/HPV+	p16-/HPV-	10.095	9.587	0.508	0.0000595	0.000395097
**PanCK-**	***TIGIT***	p16+/HPV+	p16-/HPV-	9.171	8.736	0.435	0.000114689	0.000697694
**PanCK-**	***CXCL10***	p16+/HPV+	p16-/HPV-	9.646	8.869	0.776	0.001498463	0.006836736
**PanCK-**	**HLA-DRB**	p16+/HPV+	p16-/HPV-	12.197	11.644	0.553	0.003202802	0.012305502
**PanCK-**	***CTNNB1***	p16+/HPV+	p16-/HPV-	9.965	9.808	0.157	0.006187879	0.01963979
**PanCK-**	**HLA-DQ**	p16+/HPV+	p16-/HPV-	10.207	9.682	0.525	0.006187879	0.01963979
**PanCK-**	***ITGB2***	p16+/HPV+	p16-/HPV-	10.445	9.856	0.59	0.007637609	0.022301819
**PanCK-**	***CD3E***	p16+/HPV+	p16-/HPV-	9.601	9.083	0.517	0.009873774	0.027722518
**PanCK-**	***ITGAX***	p16+/HPV+	p16-/HPV-	9.13	8.75	0.38	0.010920491	0.029525773

**PanCK-**	***PTPRC***	p16+/HPV+	p16-/HPV-	10.306	9.784	0.522	0.017773528	0.04474026
**PanCK-**	*FOXP3*	p16+/HPV+	p16-/HPV-	7.852	7.644	0.208	0.021429494	0.050463003
**PanCK-**	*TBX21*	p16+/HPV+	p16-/HPV-	8.514	8.378	0.136	0.023491539	0.053590074
**PanCK-**	*MS4A1*	p16+/HPV+	p16-/HPV-	10.099	9.39	0.708	0.041556382	0.086674739
**PanCK-**	*CD44*	p16+/HPV+	p16-/HPV-	10.832	10.667	0.165	0.078739448	0.159666103
**PanCK-**	*IDO1*	p16+/HPV+	p16-/HPV-	8.917	8.658	0.259	0.084877429	0.167460874
**PanCK-**	pan-melanocyte	p16+/HPV+	p16-/HPV-	9.268	9.089	0.179	0.125858819	0.224090092
**PanCK-**	*PSMB10*	p16+/HPV+	p16-/HPV-	9.295	9.177	0.118	0.159075644	0.263920955
**PanCK-**	*CD27*	p16+/HPV+	p16-/HPV-	9.228	8.86	0.368	0.175192036	0.284200415
**PanCK-**	*LAG3*	p16+/HPV+	p16-/HPV-	8.33	8.264	0.066	0.180826306	0.28678056
**PanCK-**	*BATF3*	p16+/HPV+	p16-/HPV-	8.416	8.328	0.088	0.19249654	0.28678056
**PanCK-**	*HAVCR2*	p16+/HPV+	p16-/HPV-	8.722	8.674	0.048	0.198535163	0.289861338
**PanCK-**	*STAT3*	p16+/HPV+	p16-/HPV-	9.421	9.325	0.096	0.224074795	0.320734511
**PanCK-**	*CTLA4*	p16+/HPV+	p16-/HPV-	8.43	8.334	0.096	0.467863376	0.60989333
**PanCK-**	*TNF*	p16+/HPV+	p16-/HPV-	8.554	8.526	0.028	0.523151625	0.670001204
**PanCK-**	*CXCR6*	p16+/HPV+	p16-/HPV-	8.257	8.253	0.004	0.593662067	0.722288848
**PanCK-**	*IFNAR1*	p16+/HPV+	p16-/HPV-	8.463	8.436	0.027	0.826912878	0.914615758
**PanCK-**	*ICAM1*	p16+/HPV+	p16-/HPV-	9.126	9.117	0.009	0.826912878	0.914615758
**PanCK-**	*BCL2*	p16+/HPV+	p16-/HPV-	8.703	8.677	0.026	0.854267173	0.917080936
**PanCK-**	*MKI67*	p16+/HPV+	p16-/HPV-	7.992	7.978	0.013	0.655545438	0.759600269
**PanCK-**	**KRT**	p16+/HPV+	p16-/HPV-	8.516	10.001	-1.486	0.00000923	0.0000842
**PanCK-**	***ITGAV***	p16+/HPV+	p16-/HPV-	8.763	9.286	-0.523	0.000163415	0.000917641
**PanCK-**	***CD276***	p16+/HPV+	p16-/HPV-	8.276	8.891	-0.615	0.000283642	0.001478989
**PanCK-**	***HIF1A***	p16+/HPV+	p16-/HPV-	9.123	9.504	-0.382	0.000758794	0.003692797
**PanCK-**	***ITGB8***	p16+/HPV+	p16-/HPV-	7.806	8.169	-0.362	0.001902732	0.008170555
**PanCK-**	***CCND1***	p16+/HPV+	p16-/HPV-	8.458	9.069	-0.611	0.002858505	0.011592825
**PanCK-**	***PTEN***	p16+/HPV+	p16-/HPV-	9.124	9.377	-0.254	0.003584495	0.013083408
**PanCK-**	***PECAM1***	p16+/HPV+	p16-/HPV-	9.231	9.593	-0.362	0.005268609	0.018314687
**PanCK-**	***VEGFA***	p16+/HPV+	p16-/HPV-	8.25	8.565	-0.314	0.006878503	0.020922113
**PanCK-**	***CD86***	p16+/HPV+	p16-/HPV-	8.172	8.427	-0.255	0.015402179	0.04015568
**PanCK-**	***EPCAM***	p16+/HPV+	p16-/HPV-	6.652	6.95	-0.298	0.019526884	0.047515418
**PanCK-**	*DKK2*	p16+/HPV+	p16-/HPV-	7.691	8.012	-0.321	0.032121645	0.071056973
**PanCK-**	*IL6*	p16+/HPV+	p16-/HPV-	7.996	8.265	-0.27	0.035038987	0.075230765
**PanCK-**	*IL12B*	p16+/HPV+	p16-/HPV-	7.704	7.894	-0.19	0.101924101	0.195801563
**PanCK-**	*VSIR*	p16+/HPV+	p16-/HPV-	8.59	8.702	-0.112	0.113394733	0.21225168
**PanCK-**	*IFNGR1*	p16+/HPV+	p16-/HPV-	8.187	8.328	-0.14	0.121590694	0.221903016
**PanCK-**	*CD47*	p16+/HPV+	p16-/HPV-	9.289	9.402	-0.113	0.153960671	0.261375092
**PanCK-**	*IL15*	p16+/HPV+	p16-/HPV-	8.181	8.329	-0.148	0.153960671	0.261375092
**PanCK-**	*GZMB*	p16+/HPV+	p16-/HPV-	8.706	8.946	-0.24	0.186594022	0.28678056
**PanCK-**	*CD274*	p16+/HPV+	p16-/HPV-	7.831	8.054	-0.222	0.19249654	0.28678056
**PanCK-**	*ARG1*	p16+/HPV+	p16-/HPV-	7.862	8.052	-0.19	0.259193639	0.357002559
**PanCK-**	*PDCD1*	p16+/HPV+	p16-/HPV-	8.717	8.84	-0.123	0.259193639	0.357002559
**PanCK-**	*PDCD1LG2*	p16+/HPV+	p16-/HPV-	7.842	7.967	-0.125	0.33161701	0.44829707
**PanCK-**	*CD4*	p16+/HPV+	p16-/HPV-	9.711	9.726	-0.016	0.358418409	0.475718979
**PanCK-**	*ICOSLG*	p16+/HPV+	p16-/HPV-	8.297	8.361	-0.064	0.581613263	0.722288848
**PanCK-**	*CMKLR1*	p16+/HPV+	p16-/HPV-	8.49	8.617	-0.127	0.642959627	0.75703311
**PanCK-**	*CD40LG*	p16+/HPV+	p16-/HPV-	8.083	8.159	-0.077	0.840565961	0.915840524
**PanCK-**	*TNFRSF9*	p16+/HPV+	p16-/HPV-	8.141	8.2	-0.059	0.881797799	0.932916512
**PanCK-**	*ITGAM*	p16+/HPV+	p16-/HPV-	8.199	8.21	-0.011	0.9790663	0.9790663

**PanCK-**	*IFNG*	p16+/HPV+	p16-/HPV-	6.008	6.033	-0.025	0.951179597	0.964390425
**PanCK-**	*LY6E*	p16+/HPV+	p16-/HPV-	9.478	9.65	-0.172	0.909472	0.93509093
**PanCK-**	*AKT1*	p16+/HPV+	p16-/HPV-	9.695	9.767	-0.072	0.895619022	0.934002694
**PanCK-**	*CSF1R*	p16+/HPV+	p16-/HPV-	7.819	7.827	-0.007	0.706842786	0.806242553
**PanCK-**	*STAT2*	p16+/HPV+	p16-/HPV-	9.57	9.649	-0.079	0.642959627	0.75703311
**PanCK-**	*FAS*	p16+/HPV+	p16-/HPV-	8.352	8.356	-0.005	0.593662067	0.722288848
**PanCK-**	***STAT1***	p16+/HPV+	p16+/HPV-	11.046	9.974	1.072	0.00000994	0.000725262
**PanCK-**	***PSMB10***	p16+/HPV+	p16+/HPV-	9.295	8.917	0.378	0.0000301	0.001100407
**PanCK-**	***CXCL10***	p16+/HPV+	p16+/HPV-	9.646	8.743	0.903	0.000658559	0.009614959
**PanCK-**	***CD40***	p16+/HPV+	p16+/HPV-	8.661	8.342	0.319	0.000658559	0.009614959
**PanCK-**	***B2M***	p16+/HPV+	p16+/HPV-	14.073	13.595	0.478	0.000977805	0.010197112
**PanCK-**	***HLA-E***	p16+/HPV+	p16+/HPV-	11.918	11.49	0.428	0.001186454	0.010826389
**PanCK-**	***STAT2***	p16+/HPV+	p16+/HPV-	9.57	9.252	0.319	0.001577486	0.011515647
**PanCK-**	***ITGB2***	p16+/HPV+	p16+/HPV-	10.445	10.028	0.418	0.001577486	0.011515647
**PanCK-**	***CD74***	p16+/HPV+	p16+/HPV-	13.894	13.304	0.59	0.001900741	0.012614009
**PanCK-**	***IDO1***	p16+/HPV+	p16+/HPV-	8.917	8.498	0.419	0.00389625	0.02370219
**PanCK-**	***CXCL9***	p16+/HPV+	p16+/HPV-	10.613	9.729	0.884	0.008285592	0.046526786
**PanCK-**	*NKG7*	p16+/HPV+	p16+/HPV-	9.149	8.867	0.282	0.011381364	0.055389305
**PanCK-**	*CD8A*	p16+/HPV+	p16+/HPV-	9.358	9.081	0.277	0.012300411	0.056120624
**PanCK-**	HLA-DRB	p16+/HPV+	p16+/HPV-	12.197	11.85	0.347	0.013284607	0.057045663
**PanCK-**	*CCL5*	p16+/HPV+	p16+/HPV-	9.746	9.449	0.296	0.041520651	0.151550376
**PanCK-**	*CD68*	p16+/HPV+	p16+/HPV-	10.095	9.871	0.224	0.047303393	0.164435605
**PanCK-**	*BATF3*	p16+/HPV+	p16+/HPV-	8.416	8.222	0.195	0.053748947	0.17834878
**PanCK-**	*TIGIT*	p16+/HPV+	p16+/HPV-	9.171	9.011	0.16	0.122148181	0.342954509
**PanCK-**	*MKI67*	p16+/HPV+	p16+/HPV-	7.992	7.896	0.096	0.150893987	0.407972632
**PanCK-**	*CD27*	p16+/HPV+	p16+/HPV-	9.228	8.941	0.288	0.175640053	0.44212841
**PanCK-**	*VSIR*	p16+/HPV+	p16+/HPV-	8.59	8.489	0.101	0.203293871	0.486321327
**PanCK-**	*CD3E*	p16+/HPV+	p16+/HPV-	9.601	9.451	0.15	0.22341259	0.494215729
**PanCK-**	*ITGAX*	p16+/HPV+	p16+/HPV-	9.13	8.95	0.18	0.244916934	0.510826749
**PanCK-**	*IFNG*	p16+/HPV+	p16+/HPV-	6.008	5.858	0.149	0.279828081	0.567429163
**PanCK-**	*AKT1*	p16+/HPV+	p16+/HPV-	9.695	9.613	0.082	0.292182449	0.576468075
**PanCK-**	*CTNNB1*	p16+/HPV+	p16+/HPV-	9.965	9.929	0.036	0.304898017	0.585725138
**PanCK-**	*LAG3*	p16+/HPV+	p16+/HPV-	8.33	8.26	0.07	0.317975553	0.59518501
**PanCK-**	*MS4A1*	p16+/HPV+	p16+/HPV-	10.099	9.742	0.357	0.331415298	0.604832918
**PanCK-**	*CSF1R*	p16+/HPV+	p16+/HPV-	7.819	7.738	0.082	0.345216948	0.614654566
**PanCK-**	*HAVCR2*	p16+/HPV+	p16+/HPV-	8.722	8.646	0.076	0.451816564	0.73294687
**PanCK-**	*CCND1*	p16+/HPV+	p16+/HPV-	8.458	8.433	0.025	0.502659503	0.764461328
**PanCK-**	*GZMB*	p16+/HPV+	p16+/HPV-	8.706	8.699	0.007	0.520255605	0.775074677
**PanCK-**	*LY6E*	p16+/HPV+	p16+/HPV-	9.478	9.412	0.067	0.574884432	0.791821953
**PanCK-**	*PTPRC*	p16+/HPV+	p16+/HPV-	10.306	10.149	0.157	0.574884432	0.791821953
**PanCK-**	*VEGFA*	p16+/HPV+	p16+/HPV-	8.25	8.215	0.035	0.632085448	0.817005761
**PanCK-**	*STAT3*	p16+/HPV+	p16+/HPV-	9.421	9.392	0.029	0.651676787	0.817005761
**PanCK-**	*CD47*	p16+/HPV+	p16+/HPV-	9.289	9.262	0.027	0.671511585	0.817005761
**PanCK-**	*CD44*	p16+/HPV+	p16+/HPV-	10.832	10.776	0.056	0.671511585	0.817005761
**PanCK-**	*BCL2*	p16+/HPV+	p16+/HPV-	8.703	8.644	0.058	0.837418002	0.922001262
**PanCK-**	*FOXP3*	p16+/HPV+	p16+/HPV-	7.852	7.843	0.009	0.923712198	0.945467946
**PanCK-**	*ICOSLG*	p16+/HPV+	p16+/HPV-	8.297	8.288	0.009	0.794928151	0.921107222
**PanCK-**	***PTEN***	p16+/HPV+	p16+/HPV-	9.124	9.579	-0.455	0.000259688	0.006319076
**PanCK-**	***PDCD1***	p16+/HPV+	p16+/HPV-	8.717	9.019	-0.302	0.000886739	0.010197112

**PanCK-**	**KRT**	p16+/HPV+	p16+/HPV-	8.516	9.249	-0.734	0.008979195	0.046820089
**PanCK-**	*CD4*	p16+/HPV+	p16+/HPV-	9.711	9.99	-0.28	0.014337781	0.058147668
**PanCK-**	*CD274*	p16+/HPV+	p16+/HPV-	7.831	8.126	-0.294	0.038861301	0.14930921
**PanCK-**	*PECAM1*	p16+/HPV+	p16+/HPV-	9.231	9.474	-0.243	0.082327724	0.261301036
**PanCK-**	*ITGAM*	p16+/HPV+	p16+/HPV-	8.199	8.441	-0.241	0.103546442	0.314953762
**PanCK-**	*ITGB8*	p16+/HPV+	p16+/HPV-	7.806	8.095	-0.289	0.11567736	0.33777789
**PanCK-**	*TNF*	p16+/HPV+	p16+/HPV-	8.554	8.658	-0.104	0.167075624	0.435590019
**PanCK-**	*CD276*	p16+/HPV+	p16+/HPV-	8.276	8.677	-0.402	0.213181952	0.486321327
**PanCK-**	*IFNAR1*	p16+/HPV+	p16+/HPV-	8.463	8.628	-0.165	0.213181952	0.486321327
**PanCK-**	*ITGAV*	p16+/HPV+	p16+/HPV-	8.763	8.998	-0.235	0.233989733	0.502389721
**PanCK-**	*CD86*	p16+/HPV+	p16+/HPV-	8.172	8.317	-0.145	0.373901959	0.649877214
**PanCK-**	*PDCD1LG2*	p16+/HPV+	p16+/HPV-	7.842	7.978	-0.136	0.38878188	0.660025051
**PanCK-**	*TNFRSF9*	p16+/HPV+	p16+/HPV-	8.141	8.228	-0.087	0.451816564	0.73294687
**PanCK-**	*HIF1A*	p16+/HPV+	p16+/HPV-	9.123	9.343	-0.22	0.485383144	0.764461328
**PanCK-**	*CTLA4*	p16+/HPV+	p16+/HPV-	8.43	8.512	-0.081	0.502659503	0.764461328
**PanCK-**	*CD40LG*	p16+/HPV+	p16+/HPV-	8.083	8.169	-0.087	0.538163957	0.785719378
**PanCK-**	*FAS*	p16+/HPV+	p16+/HPV-	8.352	8.48	-0.128	0.574884432	0.791821953
**PanCK-**	pan-melanocyte	p16+/HPV+	p16+/HPV-	9.268	9.371	-0.103	0.632085448	0.817005761
**PanCK-**	*TBX21*	p16+/HPV+	p16+/HPV-	8.514	8.577	-0.063	0.651676787	0.817005761
**PanCK-**	*IL15*	p16+/HPV+	p16+/HPV-	8.181	8.282	-0.102	0.651676787	0.817005761
**PanCK-**	*ICAM1*	p16+/HPV+	p16+/HPV-	9.126	9.224	-0.098	0.691577842	0.827625942
**PanCK-**	*IL12B*	p16+/HPV+	p16+/HPV-	7.704	7.77	-0.065	0.773900861	0.911205852
**PanCK-**	*IFNGR1*	p16+/HPV+	p16+/HPV-	8.187	8.2	-0.013	0.837418002	0.922001262
**PanCK-**	*ARG1*	p16+/HPV+	p16+/HPV-	7.862	7.922	-0.06	0.85885049	0.922001262
**PanCK-**	*IL6*	p16+/HPV+	p16+/HPV-	7.996	8.017	-0.021	0.880387508	0.931424465
**PanCK-**	HLA-DQ	p16+/HPV+	p16+/HPV-	10.207	10.207	0	0.945467946	0.945467946
**PanCK-**	*CXCR6*	p16+/HPV+	p16+/HPV-	8.257	8.282	-0.025	0.945467946	0.945467946
**PanCK-**	*DKK2*	p16+/HPV+	p16+/HPV-	7.691	7.738	-0.047	0.923712198	0.945467946
**PanCK-**	*CMKLR1*	p16+/HPV+	p16+/HPV-	8.49	8.501	-0.011	0.85885049	0.922001262
**PanCK-**	*EPCAM*	p16+/HPV+	p16+/HPV-	6.652	6.684	-0.032	0.85885049	0.922001262
**PanCK-**	***CXCL9***	p16+/HPV-	p16-/HPV-	9.729	8.787	0.942	0.000585307	0.042727409
**PanCK-**	*CCL5*	p16+/HPV-	p16-/HPV-	9.449	9.107	0.342	0.004286159	0.076568435
**PanCK-**	*CD8A*	p16+/HPV-	p16-/HPV-	9.081	8.773	0.308	0.004586319	0.076568435
**PanCK-**	*NKG7*	p16+/HPV-	p16-/HPV-	8.867	8.58	0.286	0.006392196	0.077771722
**PanCK-**	HLA-DQ	p16+/HPV-	p16-/HPV-	10.207	9.682	0.525	0.017216898	0.166540525
**PanCK-**	*TIGIT*	p16+/HPV-	p16-/HPV-	9.011	8.736	0.275	0.027133826	0.200916171
**PanCK-**	*PTEN*	p16+/HPV-	p16-/HPV-	9.579	9.377	0.201	0.030275039	0.200916171
**PanCK-**	*CD68*	p16+/HPV-	p16-/HPV-	9.871	9.587	0.284	0.039524085	0.240438182
**PanCK-**	*ITGAM*	p16+/HPV-	p16-/HPV-	8.441	8.21	0.23	0.082681606	0.315979116
**PanCK-**	*FOXP3*	p16+/HPV-	p16-/HPV-	7.843	7.644	0.199	0.086569621	0.315979116
**PanCK-**	*CD4*	p16+/HPV-	p16-/HPV-	9.99	9.726	0.264	0.086569621	0.315979116
**PanCK-**	*ITGAX*	p16+/HPV-	p16-/HPV-	8.95	8.75	0.2	0.094788167	0.329501724
**PanCK-**	*CD74*	p16+/HPV-	p16-/HPV-	13.304	13.089	0.215	0.134079246	0.407824374
**PanCK-**	*IFNAR1*	p16+/HPV-	p16-/HPV-	8.628	8.436	0.192	0.164400421	0.44776854
**PanCK-**	*CD3E*	p16+/HPV-	p16-/HPV-	9.451	9.083	0.368	0.164400421	0.44776854
**PanCK-**	*TNF*	p16+/HPV-	p16-/HPV-	8.658	8.526	0.132	0.171041102	0.44776854
**PanCK-**	*PTPRC*	p16+/HPV-	p16-/HPV-	10.149	9.784	0.366	0.184921756	0.449976272
**PanCK-**	*TBX21*	p16+/HPV-	p16-/HPV-	8.577	8.378	0.199	0.207279499	0.472856357
**PanCK-**	*CTLA4*	p16+/HPV-	p16-/HPV-	8.512	8.334	0.177	0.23153636	0.482443626

**PanCK-**	*CD274*	p16+/HPV-	p16-/HPV-	8.126	8.054	0.072	0.257743855	0.482443626
**PanCK-**	pan-melanocyte	p16+/HPV-	p16-/HPV-	9.371	9.089	0.281	0.257743855	0.482443626
**PanCK-**	*CD40*	p16+/HPV-	p16-/HPV-	8.342	8.225	0.117	0.257743855	0.482443626
**PanCK-**	HLA-DRB	p16+/HPV-	p16-/HPV-	11.85	11.644	0.206	0.276318901	0.502149015
**PanCK-**	*B2M*	p16+/HPV-	p16-/HPV-	13.595	13.462	0.133	0.326671089	0.541977033
**PanCK-**	*MS4A1*	p16+/HPV-	p16-/HPV-	9.742	9.39	0.352	0.348384601	0.552871214
**PanCK-**	*FAS*	p16+/HPV-	p16-/HPV-	8.48	8.356	0.124	0.348384601	0.552871214
**PanCK-**	*PDCD1*	p16+/HPV-	p16-/HPV-	9.019	8.84	0.179	0.370993816	0.564219761
**PanCK-**	*CTNNB1*	p16+/HPV-	p16-/HPV-	9.929	9.808	0.12	0.39449197	0.587712526
**PanCK-**	*ICAM1*	p16+/HPV-	p16-/HPV-	9.224	9.117	0.107	0.418868666	0.611548253
**PanCK-**	*ITGB2*	p16+/HPV-	p16-/HPV-	10.028	9.856	0.172	0.510867881	0.690617691
**PanCK-**	*TNFRSF9*	p16+/HPV-	p16-/HPV-	8.228	8.2	0.028	0.553304251	0.708617725
**PanCK-**	*CD44*	p16+/HPV-	p16-/HPV-	10.776	10.667	0.109	0.553304251	0.708617725
**PanCK-**	*STAT3*	p16+/HPV-	p16-/HPV-	9.392	9.325	0.067	0.627654121	0.73901211
**PanCK-**	*HLA-E*	p16+/HPV-	p16-/HPV-	11.49	11.447	0.043	0.643024372	0.745091733
**PanCK-**	*CXCR6*	p16+/HPV-	p16-/HPV-	8.282	8.253	0.029	0.771036942	0.840085026
**PanCK-**	*STAT1*	p16+/HPV-	p16-/HPV-	9.974	9.933	0.042	0.820930804	0.868520995
**PanCK-**	*CD27*	p16+/HPV-	p16-/HPV-	8.941	8.86	0.08	0.871560131	0.89611112
**PanCK-**	*PDCD1LG2*	p16+/HPV-	p16-/HPV-	7.978	7.967	0.011	0.88856505	0.900906232
**PanCK-**	*CD40LG*	p16+/HPV-	p16-/HPV-	8.169	8.159	0.01	0.991400188	0.991400188
**PanCK-**	*CCND1*	p16+/HPV-	p16-/HPV-	8.433	9.069	-0.636	0.00183958	0.067144673
**PanCK-**	*PSMB10*	p16+/HPV-	p16-/HPV-	8.917	9.177	-0.26	0.005244413	0.076568435
**PanCK-**	*VSIR*	p16+/HPV-	p16-/HPV-	8.489	8.702	-0.213	0.018251016	0.166540525
**PanCK-**	*STAT2*	p16+/HPV-	p16-/HPV-	9.252	9.649	-0.398	0.030275039	0.200916171
**PanCK-**	KRT	p16+/HPV-	p16-/HPV-	9.249	10.001	-0.752	0.043844473	0.246203582
**PanCK-**	*ITGAV*	p16+/HPV-	p16-/HPV-	8.998	9.286	-0.288	0.053687817	0.279943617
**PanCK-**	*VEGFA*	p16+/HPV-	p16-/HPV-	8.215	8.565	-0.349	0.071859823	0.315979116
**PanCK-**	*IL6*	p16+/HPV-	p16-/HPV-	8.017	8.265	-0.249	0.082681606	0.315979116
**PanCK-**	*CD47*	p16+/HPV-	p16-/HPV-	9.262	9.402	-0.14	0.086569621	0.315979116
**PanCK-**	*HIF1A*	p16+/HPV-	p16-/HPV-	9.343	9.504	-0.161	0.108275529	0.359277892
**PanCK-**	*DKK2*	p16+/HPV-	p16-/HPV-	7.738	8.012	-0.274	0.134079246	0.407824374
**PanCK-**	*EPCAM*	p16+/HPV-	p16-/HPV-	6.684	6.95	-0.266	0.177880653	0.44776854
**PanCK-**	*GZMB*	p16+/HPV-	p16-/HPV-	8.699	8.946	-0.246	0.177880653	0.44776854
**PanCK-**	*CD276*	p16+/HPV-	p16-/HPV-	8.677	8.891	-0.214	0.192166982	0.452522249
**PanCK-**	*IFNGR1*	p16+/HPV-	p16-/HPV-	8.2	8.328	-0.127	0.223236298	0.482443626
**PanCK-**	*IDO1*	p16+/HPV-	p16-/HPV-	8.498	8.658	-0.16	0.23153636	0.482443626
**PanCK-**	*IFNG*	p16+/HPV-	p16-/HPV-	5.858	6.033	-0.174	0.240053264	0.482443626
**PanCK-**	*IL12B*	p16+/HPV-	p16-/HPV-	7.77	7.894	-0.124	0.285940711	0.502149015
**PanCK-**	*AKT1*	p16+/HPV-	p16-/HPV-	9.613	9.767	-0.155	0.295786406	0.502149015
**PanCK-**	*PECAM1*	p16+/HPV-	p16-/HPV-	9.474	9.593	-0.119	0.295786406	0.502149015
**PanCK-**	*ARG1*	p16+/HPV-	p16-/HPV-	7.922	8.052	-0.13	0.359577584	0.558492844
**PanCK-**	*MKI67*	p16+/HPV-	p16-/HPV-	7.896	7.978	-0.083	0.457049089	0.641626606
**PanCK-**	*CD86*	p16+/HPV-	p16-/HPV-	8.317	8.427	-0.11	0.457049089	0.641626606
**PanCK-**	*CMKLR1*	p16+/HPV-	p16-/HPV-	8.501	8.617	-0.116	0.47019749	0.647630505
**PanCK-**	*BATF3*	p16+/HPV-	p16-/HPV-	8.222	8.328	-0.106	0.553304251	0.708617725
**PanCK-**	*LY6E*	p16+/HPV-	p16-/HPV-	9.412	9.65	-0.239	0.582523664	0.726834893
**PanCK-**	*IL15*	p16+/HPV-	p16-/HPV-	8.282	8.329	-0.047	0.597398542	0.726834893
**PanCK-**	*CSF1R*	p16+/HPV-	p16-/HPV-	7.738	7.827	-0.089	0.597398542	0.726834893
**PanCK-**	*ICOSLG*	p16+/HPV-	p16-/HPV-	8.288	8.361	-0.073	0.612443734	0.732924468

**PanCK-**	*ITGB8*	p16+/HPV-	p16-/HPV-	8.095	8.169	-0.074	0.658548953	0.7511574
**PanCK-**	*BCL2*	p16+/HPV-	p16-/HPV-	8.644	8.677	-0.033	0.754603007	0.834636659
**PanCK-**	*LAG3*	p16+/HPV-	p16-/HPV-	8.26	8.264	-0.004	0.871560131	0.89611112
**PanCK-**	*HAVCR2*	p16+/HPV-	p16-/HPV-	8.646	8.674	-0.028	0.787574295	0.84548417
**PanCK-**	*CXCL10*	p16+/HPV-	p16-/HPV-	8.743	8.869	-0.127	0.674222129	0.757203314
